# Supplementary material for: Curated and harmonised transcriptomics datasets of interstitial lung diseases
Source: Data Brief. 2025 Oct 14;63:112139. doi: 10.1016/j.dib.2025.112139 (PMC12581653; doi:10.1016/j.dib.2025.112139)
Supplement: Supplementary file 1 [file mmc1.zip › Supplementary_material/RNA-seq/fastQC reports/GSE124685/TRIMMED_SRR8397225_fastqc.html]

TRIMMED\_SRR8397225.fastq.gz FastQC Report 

FastQC Report

Sat 3 Dec 2022  
TRIMMED\_SRR8397225.fastq.gz

## Summary

- Basic Statistics
- Per base sequence quality
- Per sequence quality scores
- Per base sequence content
- Per sequence GC content
- Per base N content
- Sequence Length Distribution
- Sequence Duplication Levels
- Overrepresented sequences
- Adapter Content

## Basic Statistics

| Measure | Value |
| --- | --- |
| Filename | TRIMMED\_SRR8397225.fastq.gz |
| File type | Conventional base calls |
| Encoding | Sanger / Illumina 1.9 |
| Total Sequences | 3585481 |
| Sequences flagged as poor quality | 0 |
| Sequence length | 35-369 |
| %GC | 53 |

## Per base sequence quality

## Per sequence quality scores

## Per base sequence content

## Per sequence GC content

## Per base N content

## Sequence Length Distribution

## Sequence Duplication Levels

## Overrepresented sequences

| Sequence | Count | Percentage | Possible Source |
| --- | --- | --- | --- |
| AAGACCAACGTCAAGGCCGCCTGGGGTAAGGTCGGCGCGCACGCTGGCGA | 37215 | 1.0379360537679603 | No Hit |
| GGGCCACGAGCTGAGTGCGTCCTGTCACTCCACTCCCATGTCCCTTGGGA | 32755 | 0.9135454908281483 | No Hit |
| AAGCTCAGGGAGAGCCCTGTTAGGGCCGCCTCTGGCCCTAGTCTCAGACC | 27982 | 0.7804252762739504 | No Hit |
| ATCAAGACATGAGGGAGGCAGGGGCTCAGCTGAAGAAGCTGGTGGACACC | 24686 | 0.6884989768457844 | No Hit |
| GGGAATGTCTACGTGCGTATGCACGTGGCACTCTCTGCC | 21027 | 0.5864485127657907 | No Hit |
| CCCATAACAGCATCAGGAGTGGACAGATCCCCAAAGGACTCAAAGAACCT | 20301 | 0.5662001834621352 | No Hit |
| TCTCTGGGCTTCTATTTCGACCGCGATGATGTGGCTCTGGAAGGCGTGAG | 19966 | 0.5568569461112749 | No Hit |
| ACACTGAGGACTCTGTTCCTCCCCTTTCCGCCTAGGGGAAAGTCCCCGGA | 17783 | 0.4959725068965642 | No Hit |
| AGCTTAATGATGCTTTCTCTGGGCTTTTGGGGGAGGGTGTCCACCAGCTT | 15131 | 0.4220075353906491 | No Hit |
| GTGGTGAGGCCCTGGGCAGGCTGCTGGTGGTCTACCCTTGGACCCAGAGG | 14935 | 0.4165410442838771 | No Hit |
| ATGATGTAGCAGCAGGTGCCAGGGGCTGGCTTGTAGGCGATCAGCAGCTG | 13980 | 0.389905845268738 | No Hit |
| AGCGGTTCCGCTGCCCTGAGGCACTCTTCCAGCCTTCCTTCCTGGGCATG | 13888 | 0.38733994127984506 | No Hit |
| CCACCTTCTCCATCGGCTCCACTGGCCTCGTGGTGTATGACTACCAGCAG | 13587 | 0.37894497279444517 | No Hit |
| TCACACTTCATGATGGAGTTGAAGGTAGTTTCGTGGATGCCACAGGACTC | 12221 | 0.3408468766115341 | No Hit |
| TGGTTCCTGGCAAGCCCATGTGTGTTGAGAGCTTCTCAGACTATCCACCT | 10751 | 0.2998481933107441 | No Hit |
| AAAAGTGATGGACTGCCCATTGCTGGAGAAGACCTTCTCTCCTACTGTCA | 9647 | 0.2690573454440283 | No Hit |
| ACACCCACCGCAACTGTCTGTCTCATATCACGAACAGCAAAGCGACCCAA | 9586 | 0.26735603953834924 | No Hit |
| TCGGCTCAGCCAAACACTGTCAGGGCCCCCAGCAGGGCCTTCAGGGCCTT | 9440 | 0.26328406146901906 | No Hit |
| TCTTTCTGGCCTGGAGGCTATCCAGCGTACTCCAAAGATTCAGGTTTACT | 9287 | 0.25901685157444704 | No Hit |
| GCTGCTACAGGAGAATAGCAGACAGGTATAGTTAAGAAACAACTTTATTT | 8960 | 0.24989673630957743 | No Hit |
| CTAAGTGCTGTGTTGTCGTTCCCCCTGCTTAAAATAAAGTTGTTTCTTAA | 7627 | 0.212719018731378 | No Hit |
| ATCAAATCCTGCAGACAAGGGGAGCCCTCAGTCTGCAGGGCTCCATAATG | 7293 | 0.20340367164126652 | No Hit |
| AGCGGTTCCGGTGTCCGGAGGCGCTGTTCCAGCCTTCCTTCCTGGGTATG | 7162 | 0.19975004748316894 | No Hit |
| TCGGGCGCCCCAGACACCAGGCGTCATGGTGGGCATGGGCCAGAAGGACT | 7097 | 0.19793718053449455 | No Hit |
| TGTTCCCTCTCCTCATGAGATTGGTGAAGAAAGTATTTGGCAAAGTTCTT | 6591 | 0.18382470859558314 | No Hit |
| GGCTCCCAGAAGTGTGTGGCTGAGCTGGGTCCCCAGGCCGTGGGGGCCGT | 6458 | 0.18011530391598785 | No Hit |
| GGGTACTTCAGGGTCAGGATGCCACGCTTGCTCTGGGCCTCGTCGCCCAC | 6412 | 0.17883235192154137 | No Hit |
| AAAGCCAAGAAAACCAACGATGCCAGATACTGAATTTCTATAGCACTGAC | 6342 | 0.17688003366912278 | No Hit |
| TTTACACCTGCTAGGGTGTTCAAAGGTCAGTGCTATAGAAATTCAGTATC | 6198 | 0.1728638361212903 | No Hit |
| TCTGTCTTCTTCAGTTTCGACTTATCGAATTTCTCGATCTCAGCCATATC | 5926 | 0.1652776851976067 | No Hit |
| ACGCTCGTAGCCCTCGCGCTTCTCCTCGGCCAATTCGCGGAAGAAGTGGC | 5596 | 0.15607389915049055 | No Hit |
| AGCTTAATGATGCTTTCTCTGGGCTTTTGGGGGGAGGGTGTCCACCAGCT | 5577 | 0.15554398419626264 | No Hit |
| ACGCTCGTAGCCCTCGCGCTTCTCCTCGGCCAGTTCGCGGAAGAAGTGGC | 5400 | 0.15060740804371853 | No Hit |
| AAGACCAACGTCAAGGCCGCCTGGGGTAAGGTCGGCGCGCACGCTGGCGAGTATGGTGCGGAG | 5348 | 0.14915711448477903 | No Hit |
| GGCTATTTTTTCCATGAGCTTAATGATGCTTTCTCTGGGCTTTTGGGGGA | 5288 | 0.14748369883984883 | No Hit |
| TGAATCTTGAACTGAGTTCCACTTGTAAACTTCTTGTTTCTTGTGGTTCC | 5221 | 0.14561505136967676 | No Hit |
| TCACACTTCATGATGGAGTTGAAGGTGGTCTCGTGGATGCCGCAAGATTC | 5157 | 0.1438300746817512 | No Hit |
| TGTCCATGTCTTACTACTTTGACCGCGATGATGTGGCTTTGAAGAACTTT | 5126 | 0.14296547659853726 | No Hit |
| ACATAGCAATTCAGGAAATTTGACTTTCCATTCTCTGCTGGATGACGTGA | 5100 | 0.1422403298190675 | No Hit |
| GGGAATGTCTACGTGCGTATGCACGTGGCACTCTCT | 5082 | 0.14173830512558844 | No Hit |
| AAGGCCCACCCGTGGAGGCCAGACCCAAAGCCCACGGTACCGAGGACAGG | 4933 | 0.13758265627401176 | No Hit |
| ATGGAGTCTATGAAGTCAATGGCCTCGTTGAACCAGGAGCTGATGTCTGC | 4730 | 0.13192093334199792 | No Hit |
| TACATCAAAGATTACATGAAATCAATCAAAGGGAAACTTGAAGAACAGAG | 4578 | 0.12768161370817474 | No Hit |
| ACACTGAGGACTCTGTTCCTCCCCTTTCCGCCTAGGGAAAGTCCCCGGAC | 4529 | 0.1263149909314817 | No Hit |
| AAAGAAGAACATGTGATCATCCAGGCCGAGTTCTATCTGAATCCTGACCA | 4510 | 0.1257850759772538 | No Hit |
| AAGACCAACGTCAAGGCCGCCTGGGGTAAGGTCGGCGCGCACGCTGGCGAGT | 4457 | 0.12430689215756548 | No Hit |
| GGCCTTCTTCTGGCGCAGAGGAAGCAGGCGCATCTGGGTGTGGGCAATGA | 4448 | 0.12405587981082596 | No Hit |
| TTTTGAGGGTCACTACCAGTACAAGAGCATCCCTGTGGAGGACAACCACA | 4299 | 0.11990023095924927 | No Hit |
| TGAGTTGGCCAGAACTCTGAAAAGATTGGGAATGGATGGCTACAGGGGAA | 4241 | 0.11828259583581674 | No Hit |
| GACAGACTCCAGCTTTGAAGGACTTTCCAGAGCCTTCCACAGCCCAAGGT | 4076 | 0.11368070281225866 | No Hit |
| ACACTGAGGACTCTGTTCCTCCCCTTTCCGCCTAGGGAAAGGTACCCGGC | 4001 | 0.11158893325609591 | No Hit |
| ATTCGAGGGCGGATCCTCTCTGGCGTGGTGACCAAGATGAAGATGCAGAG | 3981 | 0.11103112804111917 | No Hit |
| TTGGTGGTGGGGAAGGACAGGAACATCCTCTCCAGGGCCTCCGCACCATACTCGCCAGCGTGCGC | 3922 | 0.10938560265693781 | No Hit |
| TTGGTGGTGGGGAAGGACAGGAACATCCTCTCCAGGGCCTCCGCACCATA | 3866 | 0.10782374805500293 | No Hit |
| CGGTTGTACTTGCGGATGTAGTGCAGATAGTCTCGGCGGATGACAATGGT | 3831 | 0.10684758892879366 | No Hit |
| TACTCGTGCGCCTCGCTTCGCTTTTCCTCCGCAACCATGTCTGACAAACC | 3813 | 0.1063455642353146 | No Hit |
| TTTGTTCACACCAACTTGCGCAAAAACAACAGACAGCCCTATGCTGTCAG | 3734 | 0.10414223363615649 | No Hit |
| GAAAAGTTTGGAAGAGGCAGAGAAATCCTGCTCTCCTCGCCTTCCAGCAG | 3675 | 0.10249670825197511 | No Hit |
| TGAGGCGGAGCACCAGAGAGCCTACCTGGAAGACACATGCGTGGAGTGGC | 3622 | 0.10101852443228677 | No Hit |
| TTGGTGGTGGGGAAGGACAGGAACATCCTCTCCAGGGCCTCCGCACCATACTCGCCAGCGTGCGCGCCGACTACC | 3618 | 0.10090696338929143 | No Hit |

## Adapter Content

Produced by FastQC (version 0.11.7)
